# A Multi-Centre Prospective Study of the Efficacy and Safety of Alglucosidase Alfa in Chinese Patients With Infantile-Onset Pompe Disease

**DOI:** 10.3389/fphar.2022.903488

**Published:** 2022-06-27

**Authors:** Diqi Zhu, Jiacong Zhu, Wenjuan Qiu, Benzhen Wang, Lin Liu, Xiaodan Yu, Zhenheng Ou, Guangsong Shan, Jian Wang, Bin Li, Xiaokang Chen, Cong Liu, Zipu Li, Lijun Fu

**Affiliations:** ^1^ Department of Cardiology, Shanghai Children’s Medical Center, Shanghai Jiao Tong University School of Medicine, Shanghai, China; ^2^ Department of Pediatrics, The Second Hospital of Jiaxing, Jiaxing, China; ^3^ Department of Pediatric Endocrinology and Genetic Metabolism, Xinhua Hospital, Shanghai Institute of Pediatric Research, Shanghai Jiao Tong University School of Medicine, Shanghai, China; ^4^ Heart Center, Women and Children’s Hospital, Qingdao University, Qingdao, China; ^5^ Department of Pediatric Cardiology, Shenzhen Children’s Hospital, Shenzhen, China; ^6^ Department of Developmental and Behavioral Pediatrics, Shanghai Children’s Medical Center, Shanghai Jiao Tong University School of Medicine, Shanghai, China; ^7^ Research Division of Birth Defects, Institute of Pediatric Translational Medicine, Shanghai Children’s Medical Center, Shanghai Jiao Tong University School of Medicine, Shanghai, China; ^8^ Medical Department, Sanofi Investment Co., Ltd., Shanghai, China; ^9^ Shanghai Clinical Research Center for Rare Pediatric Disease, Shanghai, China

**Keywords:** Pompe disease, glycogen storage disease type II, enzyme replacement therapy, alglucosidase alfa, survival rate, left ventricular mass index

## Abstract

**Background:** A high prevalence of infantile-onset Pompe disease (IOPD) in the Chinese population has been noted, but there are currently no reported clinical trials of enzyme replacement therapy (ERT) for IOPD in this population**.** The purpose of this study was to evaluate the efficacy and safety of alglucosidase alfa in Chinese patients with IOPD.

**Materials and Methods:** A multicentre, single-arm, prospective, open-label clinical trial was performed at 4 sites in China. Eligible Chinese subjects with IOPD received an infusion of alglucosidase alfa at a dose of 20 mg/kg every 2 weeks for up to 52 weeks. The primary endpoints of clinical efficacy were the survival rate and changes in the left ventricular mass index (LVMI). The safety assessment was based on the incidence of adverse events (AEs).

**Results:** A total of 10 eligible subjects were enrolled in the study. The mean age at the start of ERT was 5.36 ± 1.56 months. Nine subjects had survived after 52 weeks of treatment. One subject discontinued the study and died after mechanical ventilation was withdrawn. The intent-to-treat analysis demonstrated that the survival rate was 90.0% (95% confidence interval: 55.5–99.7%). The mean LVMI at week 52 was 70.59 ± 39.93 g/m^2^ compared to that of 298.02 ± 178.43 g/m^2^ at baseline, with a difference of -227.60 ± 155.99 g/m^2^. All subjects had left ventricular mass (LVM) Z scores >10 at baseline, and eight subjects (80%) achieved Z scores <5 at week 52. No treatment-related AEs were observed, and no AEs led to the discontinuation of treatment.

**Conclusions:** This clinical trial is the first study of ERT for IOPD in China, indicating that alglucosidase alfa has favourable efficacy and safety for the treatment of Chinese patients with IOPD (ClinicalTrials.gov number, NCT03687333).

## Introduction

Pompe disease, also called acid maltase deficiency or type II glycogen storage disease, is a rare, autosomal recessive, but lethal metabolic myopathy caused by mutations in the gene coding for acid alpha-glucosidase (GAA), the enzyme that decomposes glycogen in the lysosome (Kohler et al., 2018). With GAA deficiency, a lysosomal accumulation of glycogen develops in multiple tissues, with skeletal and cardiac muscles being the most severely affected.

Two categories of Pompe disease, namely, infantile-onset Pompe disease (IOPD) and late-onset Pompe disease (LOPD), are recognized according to the age of symptom onset and the occurrence of cardiomyopathy. Classic IOPD is the most severe form of Pompe disease, which is characterized by an age of onset of less than 12 months, rapidly progressive hypertrophic cardiomyopathy and respiratory distress (Kishnani et al., 2006b; van den Hout et al., 2003). Most untreated IOPD patients cannot survive beyond 1 year of age (Kishnani et al., 2006b). In contrast, LOPD manifests after 12 months of age and generally lacks significant cardiac involvement. The incidence of Pompe disease appears to vary by ethnicity and geographic region. The estimated frequency of IOPD is approximately 1 in 100,000; higher frequencies of the infantile-onset form have been reported in some populations, including African Americans (approximately 1 in 14,000) and persons with Chinese ancestry (1 in 50,000 to 1 in 40,000) (Hirschhorn R, 2001).

Enzyme replacement therapy (ERT) using recombinant human GAA (rhGAA) has changed the natural course of Pompe disease. The lifespan of infants with IOPD has been significantly extended. Sustained and striking cardiac improvement has been observed in the majority of patients(Kishnani et al., 2007). The safety and efficacy of alglucosidase alfa have been demonstrated in a number of clinical trials (Kohler et al., 2018); however, these trials did not include Chinese patients.

rhGAA has been approved in the USA and Europe since 2006, and in China since 2015, since the National Medical Products Administration in China considered it to be urgently needed in clinical practice. Here, we report the results of a multicentre prospective study of alglucosidase alfa in China, which was the first clinical trial to evaluate the efficacy and safety of alglucosidase alfa in Chinese patients with IOPD.

## Materials and Methods

### Study Design

This study was conducted in accordance with consensus ethics principles derived from international ethics guidelines, including the Declaration of Helsinki, the International Conference on Harmonization guidelines for Good Clinical Practice, and all applicable laws, rules, and regulations. The protocol and its amendments were reviewed and approved by independent ethics committees and/or institutional review boards.

This was a multicentre, single-arm, prospective, open-label clinical trial to assess the efficacy and safety of a 52-week treatment with alglucosidase alfa (Myozyme^®^) in subjects with IOPD at 4 sites (Shanghai Children’s Medical Center, Xinhua Hospital Affiliated to Shanghai Jiao Tong University School of Medicine, Shenzhen Children’s Hospital, and Qingdao Women and Children’s Hospital) in China. The eligible subjects with IOPD received an infusion of alglucosidase alfa at a dose of 20 mg/kg every 2 weeks for up to 52 weeks. Subjects received treatment with alglucosidase alfa for 52 weeks or withdrew due to intolerable side effects of the study drug or death, whichever came first. Since GAA protein levels are unable to be detected in China, a negative cross-reactive immunologic material (CRIM) status was rapidly inferred by gene mutation analysis. Any patient who carried 2 predicted null alleles was considered CRIM-negative (Bali et al., 2012). The null allele is a nonsense or frameshift mutation resulting in a premature termination codon in any exon but the last or multiexon deletion. If a patient had at least one missense mutation, he or she was considered CRIM-positive. For patients whose CRIM status was inferred to be negative by gene mutation, an immune tolerance induction (ITI) regimen including rituximab, methotrexate, and intravenous immune globulin was recommended concomitantly with ERT (Mendelsohn et al., 2009; Messinger et al., 2012; Banugaria et al., 2013; Kazi et al., 2017).

IOPD is a rapidly fatal disorder, and several clinical trials have shown that treatment with alglucosidase alfa can improve survival, cardiac and respiratory function, growth, and motor development in severely affected infants(Kishnani et al., 2007; Nicolino et al., 2009; Kishnani et al., 2006b). It was considered unethical to include a placebo group as part of the study design. Thus, no control group was applied for this study.

### Patients

All eligible patients had a confirmed diagnosis of IOPD with the documented onset of Pompe disease symptoms up to 12 months of age (corrected for gestation if born before 40 weeks) and a diagnosis of Pompe disease confirmed by GAA enzyme deficiency from any tissue source and *GAA* gene mutations. The pathogenicity of mutations was determined according to the Pompe disease *GAA* variant database (https://www.pompevariantdatabase.nl). The exclusion criteria were patients who had previously been treated with GAA, patients who were participating in another clinical study using any investigational therapy, patients with conditions/situations such as clinical signs of cardiac failure with a left ventricular ejection fraction (LVEF) < 40%, respiratory insufficiency (O_2_ saturation <90% or CO_2_ partial pressure >55 mm Hg [venous] or >40 mm Hg [arterial] in room air or with any ventilator use), patients who were dependent on invasive or noninvasive ventilator support, patients with major congenital anomalies or clinically significant intercurrent organic diseases unrelated to Pompe disease, patients who were not suitable for participation, regardless of the reason, as judged by the investigator, including those with medical or clinical conditions, and patients who were potentially at risk of noncompliance with the study procedures. The dates for the first eligible subject enrolled and last eligible subject to complete the study were December 4, 2018, and December 30, 2020, respectively.

### Assessments of Clinical Efficacy

The primary endpoints of clinical efficacy were the survival rates and changes from baseline in the left ventricular mass index (LVMI) at 52 weeks of treatment. The secondary endpoints were survival free of invasive ventilator use at 52 weeks of treatment; survival free of any ventilator use at 52 weeks of treatment; physical growth (change from baseline at week 52 regarding length and weight); the number of motor development milestones achieved at week 52 and changes from baseline; changes from baseline in the GESELL Developmental Scale score at week 52; and the proportion of subjects with signs and/or symptoms of cardiac failure at week 52.

### Safety Assessments

Safety was evaluated in terms of adverse events (AEs) reported by the caregiver or noted by the investigator. Clinical and laboratory safety assessments included clinical haematology, chemistry, and urinalysis assessments. Other safety endpoints included vital signs (body temperature, heart rate, respiratory rate, and blood pressure), physical examinations, and electrocardiograms.

### Statistical Analysis

Available clinical parameters and demographics were described. Efficacy and safety were analysed in the intent-to-treat (ITT) population defined as all patients treated with alfa glucosidase. At the end of the study, the proportion of subjects who were alive was calculated, and the changes in LVMI and LVM Z scores from baseline were analysed. The number of subjects who were alive independent of an invasive ventilator or any ventilator, physical growth, the number of motor development milestones achieved, motor development gains (GESELL Developmental Scale), and the proportion of patients with signs/symptoms of heart failure at each visit were calculated or statistically described. The safety assessment was based on the incidence of AEs.

## Results

### Patient Characteristics

A total of 10 subjects were enrolled in the study, and all subjects (100.0%) were included in the ITT population and safety population. The mean age at the start of ERT was 5.36 ± 1.56 (range from 1.5 to 7.4) months. Of the 10 subjects, four (40.0%) were male and six (60.0%) were female; all subjects were predominantly of Asian descent (100.0%), and nine subjects (90.0%) were of Han nationality. Seven subjects (70%) had a medical history other than IOPD, and one subject (10%) had a surgical history. The average length and weight of all subjects were 65.16 ± 6.31 cm and 5.76 ± 1.02 kg, respectively. The mean age at disease onset was 4.2 ± 1.48 months. Various *GAA* gene mutations were found in the subjects, including missense, nonsense, insertion, splicing, and duplication mutations. The most frequent mutation observed in our study was c.1935C > A, with four out of ten patients having this mutation. Nine subjects (90%) had a CRIM-positive status, and one (10%) subject had a negative status. The mean LVMI and LVEF were 298.02 ± 178.43 g/m^2^ and 57.97 ± 13.32%, respectively. No subject was dependent on a ventilator at the beginning of this study. The baseline characteristics of the study subjects are shown in Table 1.

**TABLE 1:** Baseline characteristics of 10 Chinese patients with infantile-onset Pompe disease.

Patient	^a^Age (months)	Sex	Ethnicity	Length (cm)	Weight (kg)	Onset Age (months)	^b^Medical/Surgical History	GAA Gene mutations	^c^CRIM Status	^d^GAA Activity (Normal Reference Range)	LVMI (g/m^2^)	LVEF (%)	Ventilator use
0101	6.1	F	Han	70.5	6.3	5	Yes	c.258dupC, p.Asn87Glnfs*9(het) c.1987C > T, p.Gln663*(het)	−	1.12 (2.88–98.02 μmol/L/h)	296.5	76.7	No
0102	1.5	F	Han	55	3.9	1	No	c.1802C > T, p.Ser601Leu(het) c.1822C > T, p.Arg608*(het)	+	0.76 (2.88–98.02 μmol/L/h)	79.0	63.9	No
0103	5.8	F	Han	58	5.0	4	No	c.1935C > A, p.Asp645Glu (het) c.2662G > T, p.Glu888*(het)	+	1.05 (2.88–98.02 μmol/L/h)	291.1	61.1	No
0104	7	F	She	64	6.2	6	Yes	c.1432G > A, p.Gly478Arg(het) c.1935C > A, p.Asp645Glu(het)	+	0.37 (2.88–98.02 μmol/L/h)	745.5	49.2	No
0105	5.9	F	Han	65.6	4.6	5	Yes	c.1832G > A, p.Gly611Asp(het) c.1935C > A, p.Asp645Glu(het)	+	1.10 (2.88–98.02 μmol/L/h)	298.8	41.1	No
0106	5.5	M	Han	77	6.6	4	No	c.796C > T, p.Pro266Ser(het) c.1562A > T, p.Glu521Val(het)	+	0.03 (2.88–98.02 μmol/L/h)	228.4	45.2	No
0302	5.4	M	Han	65	6.3	4	Yes	c.1935C > A, p.Asp645Glu(het) c.2662G > T, p.Glu888*(het)	+	6.8 (24.8–93.0 nmol/g/min)	361.9	79.8	No
0303	4.8	M	Han	64.5	7.0	4	Yes	c.1082C > T, p.Pro361Leu(het) c.1942G > A, p.Gly648Ser(het)	+	2.06 (>14nmol/1 h/mg)	140.3	58.7	No
0401	7.4	M	Han	70	6.5	6	Yes	c.1121G > A, p.Cys374Tyr(het) c.1935C > A, p.Asp645Glu(het)	+	2.4 (62.3–301.7 nmol/h/mgPr)	231.8	44.0	No
0403	4.2	F	Han	62	5.2	3	Yes	c.859-2A > T, p.?(het) c.1861T > G, p.Trp621Gly(het)	+	0.7 (62.3–301.7 nmol/h/mgPr)	306.9	60.0	No

F, female; M, male; w/s, with support.

a Age at first infusion.

b Other than IOPD.

c CRIM status was predicted by *GAA* gene mutations.

d All patients had GAA enzyme test results below the lower limit of the normal reference range.

Due to the different GAA enzyme detection methods used in the medical center of each patient, the normal reference value range and numerical unit of each patient in the table are different. LVEF: left ventricular ejection fraction. LVMI: left ventricular mass index.

### Efficacy

#### Survival Rate and Ventilator Independence

In the ITT population, among all 10 patients, nine subjects survived, and one subject died. The survival rate was 90.0%, and the 95% confidence interval (CI) was 55.5–99.7%. The subject who died (Patient 0101 in Table 1) was the only CRIM-negative subject in this study. She suffered from respiratory failure and received invasive mechanical ventilation for nearly one month. She was withdrawn from the study when her family members signed to stop all treatment and died after mechanical ventilation was withdrawn.

Invasive or noninvasive ventilator dependency was continuously monitored during this study. In the ITT population, one subject (Patient 0101 in Table 1) used an invasive ventilator during this study, as described above. Another subject had been supported temporarily by a noninvasive ventilator at week 2. The remaining eight subjects did not use any ventilators. At the end of the study, all nine survivors were free from the use of any ventilators. Thus, the survival rate was 100% (*n* = 9; 95% CI: 66.4–100%) in the subjects who did not use any invasive ventilators and 100% (*n* = 8; 95% CI: 63.1–100%) in the subjects who did not use any ventilators at week 52 (Table 2).

**TABLE 2:** Survival under different ventilation conditions at week 52.

	Free of Any Ventilator use (*n* = 8)	Free of Invasive Ventilator use (*n* = 9)
Survival, n (%)	8 (100%)	9 (100%)
95% confidence interval	(63.1%, 100%)	(66.4%, 100%)

#### Cardiac Improvement

**FIGURE 1:**
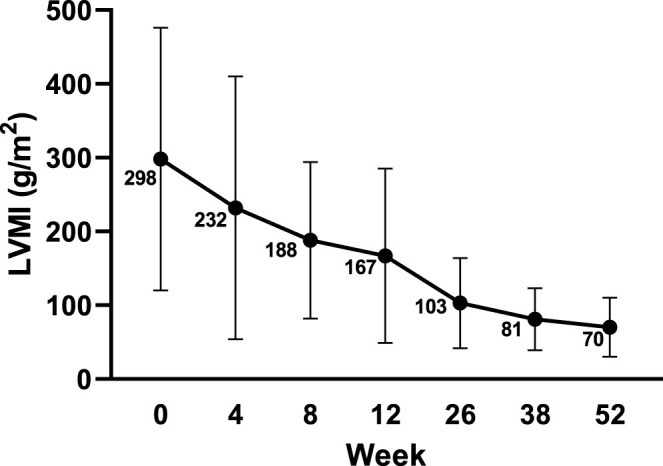
LVMI changes from baseline echocardiography by visit. Data are presented as the mean ± SD. LVMI: left ventricular mass index.

**FIGURE 2:**
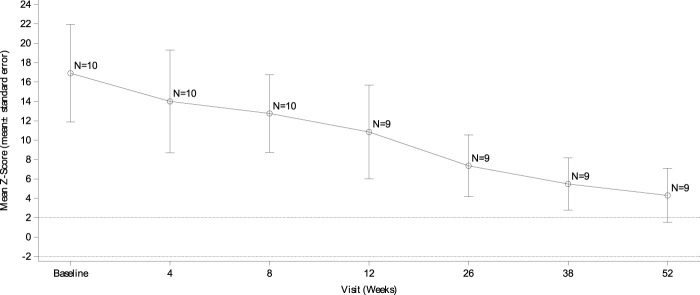
LVM Z score changes from baseline echocardiography by visit. Data are presented as the mean ± SD. LVM: left ventricular mass.

At baseline, all 10 subjects (100%) were found to have left ventricular hypertrophy (abnormally elevated LVMI scores and LVM Z scores >2). The mean LVMI of the subjects decreased steadily from baseline over the study period. The mean LVMI was 70.59 ± 39.93 g/m^2^ at week 52 compared to that of 298.02 ± 178.43 g/m^2^ at baseline, and the difference was −227.60 ± 155.99 g/m^2^ (Figure 1). The Z score of the LVM also improved gradually. The mean LVM Z score was 16.63 ± 5.17 at baseline and 4.30 ± 2.78 at week 52 (Figure 2). Compared to 100% of subjects with LVM Z scores >10 at baseline, eight subjects (80%) had LVM Z scores <5, with one subject (10.0%) having an LVM Z score <2 at week 52. Before she passed away, the LVM Z score of the subject who died decreased from 12.22 at baseline to 8.90 at week 8, suggesting the effect of the treatment.

During the study, two subjects (20.0%) at week 4 and one subject (10.0%) at week 8 had signs and/or symptoms of cardiac failure. No subject exhibited any signs and/or symptoms of cardiac failure at week 52.

#### Physical Growth and Other Developments

Overall, the length and weight of the subjects increased in this study. The mean baseline length and weight were 65.16 ± 6.31 cm and 5.76 ± 1.02 kg, respectively. At week 52, the differences in length and weight from baseline were 12.66 ± 4.68 cm and 2.69 ± 0.75 kg, respectively. Age-equivalent weight and length values were also analysed. At baseline, nine (9/10, 90.0%) and seven (7/10, 70.0%) patients had lengths/weights above or equal to the 3rd percentile of their age-appropriate lengths/weights, respectively. At week 52, there were seven subjects (7/10, 70%) whose length was greater than or equal to the 3rd percentile. In terms of weight, five subjects (5/10, 50%) reached standard weight-for-age percentiles.

In the ITT population, all subjects obtained new motor development milestones during the treatment. At baseline, six subjects (6/10, 60%) presented at least one of the following motor development milestones: five subjects could hold their head up, one subject could bear weight on their legs, one subject could roll, and one subject could sit with support. At week 52, all nine survivors achieved one or more motor development milestones: all subjects could hold their head up and sit with support, eight subjects could roll over, seven subjects could sit without support, three subjects could bear weight on their legs, three subjects could walk with support, two subjects could pull to stand, one subject could walk without support, and one subject could walk up steps. No subject ever learned to run or walk down steps during the treatment period. At baseline, the number of motor development milestones achieved was 0.8 ± 0.92, compared to that of 4.8 ± 1.99 at week 52, with a difference of 3.9 ± 1.62.

The measured mature month ages for motor, adaptive (cognitive), language, and personal-social behaviours based on the GESELL Developmental Scale increased during the trial. The mean changes from baseline were 5.87 ± 3.68, 8.50 ± 3.26, 6.82 ± 3.05, and 8.73 ± 3.11 at week 52, respectively.

### Safety

In the safety population, at least one AE was reported for each subject. All AEs identified in this study were treatment-emergent adverse events (TEAEs), and at least one serious treatment emergent adverse event (SAE) was reported for nine subjects (90%). The most commonly reported SAEs (≥20%) were pneumonitis (60.0%; *n* = 6), followed by pneumonia (30.0%; *n* = 3), respiratory tract infection (20.0%; *n* = 2), gastroenteritis rotavirus (20.0%; *n* = 2) and bronchitis (20.0%; *n* = 2). No SAEs were related to the study drug, and the outcome of most SAEs was recovered or resolved. No SAEs led to discontinuation from the study. The infusion-associated reactions, such as rash, fever, and urticaria, were not common. In our study, one subject (Patient 0101 in Table 1) with a predicted CRIM-negative status had an AE (asphyxia) leading to death, which was unrelated to the study drug.

## Discussion

In this first clinical trial for Chinese patients with IOPD, we report that 90.0% (9/10) of the patients who received alglucosidase alfa at a dose of 20 mg/kg every 2 weeks from the age of 1.5–7.4 (5.36 ± 1.56) months had survived after 52 weeks of treatment. In contrast, a multinational study of the natural course of IOPD showed that just a few percent (25.7%) of patients without treatment are able to survive beyond one year of age (Kishnani et al., 2006a). In our previous study of the clinical course of IOPD, 17 untreated Chinese patients died at a median age of 8.2 months. Only two of them survived beyond one year of age (Fu et al., 2014). Chen *et al* investigated the clinical outcomes of 25 Chinese patients with IOPD. Only one patient receiving ERT survived beyond the age of two years. Of the remaining 24 patients who were not treated with ERT, all but one patient died at a median age of 8.3 months (Chen et al., 2017). Our current study clearly demonstrated the ability of alglucosidase alfa to change the natural course of disease in Chinese IOPD patients and extend their lifespans. This finding is similar to the results of a US clinical trial of ERT for IOPD in which a 52-week alglucosidase alfa treatment reduced the risk of death by 99% compared to the untreated historic cohort (Kishnani et al., 2007).

Cardiac and respiratory failure is the main cause of death in the absence of treatment for IOPD patients. Most subjects (90%) in this study survived independent of the use of invasive ventilators and any other ventilators after 52 weeks of treatment, although one subject needed a noninvasive ventilator and one subject needed an invasive ventilator during the study. The ventilator-free survival rate by the end of the 52-week treatment period was higher than that (66.7%) in the US clinical trial mentioned above (Kishnani et al., 2007). In our trial, a remarkable improvement in cardiac status was observed after ERT therapy. One hundred percent of the surviving subjects had no symptoms or signs of cardiac failure at the end of the clinical trial, which likely contributed to the increased survival rates observed in this study. Moreover, the mean LVMI and LVM Z scores of the ITT population gradually decreased during treatment. Eight subjects (80%) achieved an LVM Z score <5, and one subject (10%) had a Z score <2 after 52 weeks of alglucosidase alfa administration. In comparison, Nicolino *et al* reported that more than half of IOPD patients (57%, 12/21) attained a normal LVM score (Z score <2) with a median treatment duration of 120 weeks (Nicolino et al., 2009). The difference in the regression of cardiac hypertrophy observed in the two studies may be attributed to the different durations of ERT therapy. Long-term alglucosidase alfa treatment will lead to further improvements in left ventricular hypertrophy in patients with IOPD.

For untreated IOPD patients, physical growth and motor development are severely delayed, and major developmental milestones, such as rolling over, sitting or standing, are not generally achieved. Among the 18 patients in the US clinical trial, 15 maintained normal physical growth, 13 had consistent motor and functional gains, and all acquired cognitive, language, and personal/social development skills during the 52-week treatment period (Kishnani et al., 2007). In our study, a total of 3.9 ± 1.62 motor development milestones were achieved at 52 weeks compared with the baseline. At week 52, the length and weight of the patients increased from baseline by 12.66 ± 4.67 cm and 2.69 ± 0.75 kg, respectively. Seven subjects (70%) and five subjects (50%) remained in percentiles greater than or equal to the 3rd percentile at the end of the study. Although our findings for length and weight were lower than those of previous studies, the length and weight of the subjects showed the same increasing trends as those reported in the published literature (Kishnani et al., 2007; Nicolino et al., 2009). In this study, the GESELL Developmental Scale was used to evaluate the functional skills of IOPD patients, which indicated remarkable progress in comprehensive development after alglucosidase alfa treatment. Taken together, the secondary points achieved in our study were similar to those of the US clinical trial.

At least one TEAE was observed for each subject in this study, such as vomiting, otitis media, pneumonia, and upper respiratory tract infection. However, the majority of AEs reported in previous studies were related to rhGAA intravenous infusion, such as rash, fever, and urticaria (Kishnani et al., 2007; Nicolino et al., 2009), which were not common in our study. This might be attributed to the pretreatment with 5 mg of dexamethasone 30 min prior to the infusion of alglucosidase alfa in the majority of patients in our cohort. No treatment related TEAEs were observed in our study, and no AEs led to the discontinuation of treatment, indicating the good tolerability and good safety of alglucosidase alfa administration. One patient died in our study; this patient was predicted to have a CRIM-negative status. Owing to a complete inability to generate native enzymes, CRIM-negative patients are more likely to develop high and sustained anti-rhGAA IgG antibody titers, rendering ERT ineffective (Kishnani et al., 2010; Banugaria et al., 2011; Berrier et al., 2015). Identifying CRIM status is crucial before initiating ERT because ITI has been found to be effective in CRIM-negative patients before or shortly after the start of ERT (Bali et al., 2012). A patient is designated to be CRIM-negative if the GAA protein is undetectable (Kishnani et al., 2010). Unfortunately, CRIM status cannot be tested in China. Bali *et al.* determined that types of *GAA* gene mutations are well associated with the levels of GAA protein. Most CRIM-negative patients have two null alleles (frame shift, nonsense, and multiexon deletions), resulting in the lack of production of the GAA protein (Bali et al., 2012). The only patient (Patient 0101 in Table 1) who died in this study had a frameshift mutation (c.258dupC, p. Asn87Glnfs*9) and a nonsense mutation (c.1987C > T, p. Gln663*) in the *GAA* gene, causing two null alleles. This patient’s CRIM status was accordingly predicted to be negative. ITI with methotrexate, rituximab, and intravenous immunoglobulin was administered for this patient, followed by ERT. However, ERT did not produce the expected effects in this patient. Very recently, Li *et al* reported that in CRIM-negative IOPD patients treated with ITI + ERT, the clinical outcomes of the early group (treatment initiation at age ≤4 weeks) were much better than those of the intermediate group (treatment initiation at age >4 and ≤15 weeks) and late group (treatment initiation at age >15 weeks) (Li et al., 2021). In the intermediate group and late group, 66% (10/15) of the patients needed a breathing device during the treatment. This investigation indicates that the age at ITI + ERT treatment initiation markedly impacts the clinical outcomes of CRIM-negative IOPD patients. The CRIM-negative patient in our study was older than 15 weeks at initial treatment, which might be the reason why the patient did not achieve the expected outcome even after receiving ITI + ERT therapy. This highlights the significance of the early recognition of IOPD patients and the early application of ITI + ERT in CRIM-negative patients.

Since it received approval in Europe and the USA in 2006, alglucosidase alfa has shown good efficacy, safety, and tolerability in IOPD patients. However, data to evaluate the efficacy, safety, and tolerability of alglucosidase alfa for Chinese IOPD patients were not available until the current study was performed. Our clinical trial is the first study of ERT in China, and it demonstrated that alglucosidase alfa has favourable efficacy and safety for treating Chinese patients with IOPD.

## Data Availability

The original contributions presented in the study are included in the article/Supplementary materials, further inquiries can be directed to the corresponding authors.

## Abbreviations

AEs, Adverse events; CRIM, Cross-reactive immunologic material; ERT, Enzyme replacement therapy; GAA, Acid alpha-glucosidase; ITT, Intent-to-treat; IOPD, Infantile-onset Pompe disease; LOPD, Late-onset Pompe disease; LVMI, Left ventricular mass index; LVM, Left ventricular mass; rhGAA, Recombinant human GAA; SAEs, Serious treatment-emergent adverse events; TEAEs, Treatment-emergent adverse events; ITI, Immune tolerance induction,
